# Supplementary material for: The dynamic lateral gate of the mitochondrial β-barrel biogenesis machinery is blocked by darobactin A
Source: Nat Commun. 2025 Nov 20;16:11349. doi: 10.1038/s41467-025-66417-0 (PMC12728192; doi:10.1038/s41467-025-66417-0)
Supplement: Supplementary file 1 — Supplementary Information [file 41467_2025_66417_MOESM1_ESM.pdf]

Supplementary Information

The dynamic lateral gate of the mitochondrial β-barrel biogenesis machinery is blocked by darobactin A

Kathryn A. Diederichs, Istvan Botos, Scout Hayashi, Gvantsa Gutishvili, Vadim Kotov, Katie Kuo, Akira Iinishi, Gwendolyn Cooper, Benjamin Schwarz, Herve Celia, Thomas C. Marlovits, Kim Lewis, James C. Gumbart, Joseph A. Mindell, Susan K. Buchanan

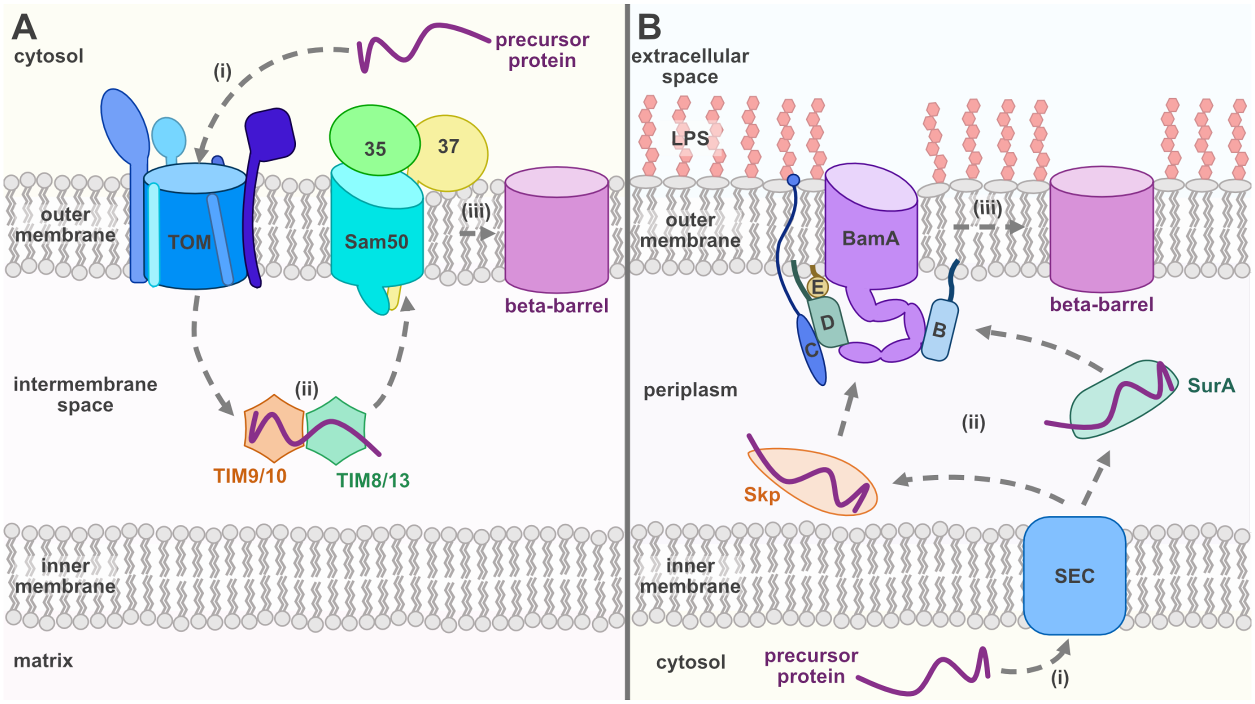

Supplementary Figure 1. Comparison of β-barrel protein import and assembly in mitochondria and Gram-negative bacteria. (A) Mitochondrial outer membrane β-barrel proteins are synthesized in the cytosol and translocated into the intermembrane space by the TOM complex (i), small TIM chaperones assist transport of the precursor protein to the SAM complex (ii). The SAM complex facilitates the folding and insertion of the β-barrel protein into the outer membrane (iii). (B) Gram-negative bacterial outer membrane β-barrel precursor proteins are synthesized in the cytosol, translocated into the periplasm via the SEC translocon (i), bound by periplasmic chaperones and transported to the BAM complex (ii). The BAM complex facilitates the folding and insertion of the β-barrel protein into the outer membrane (iii).

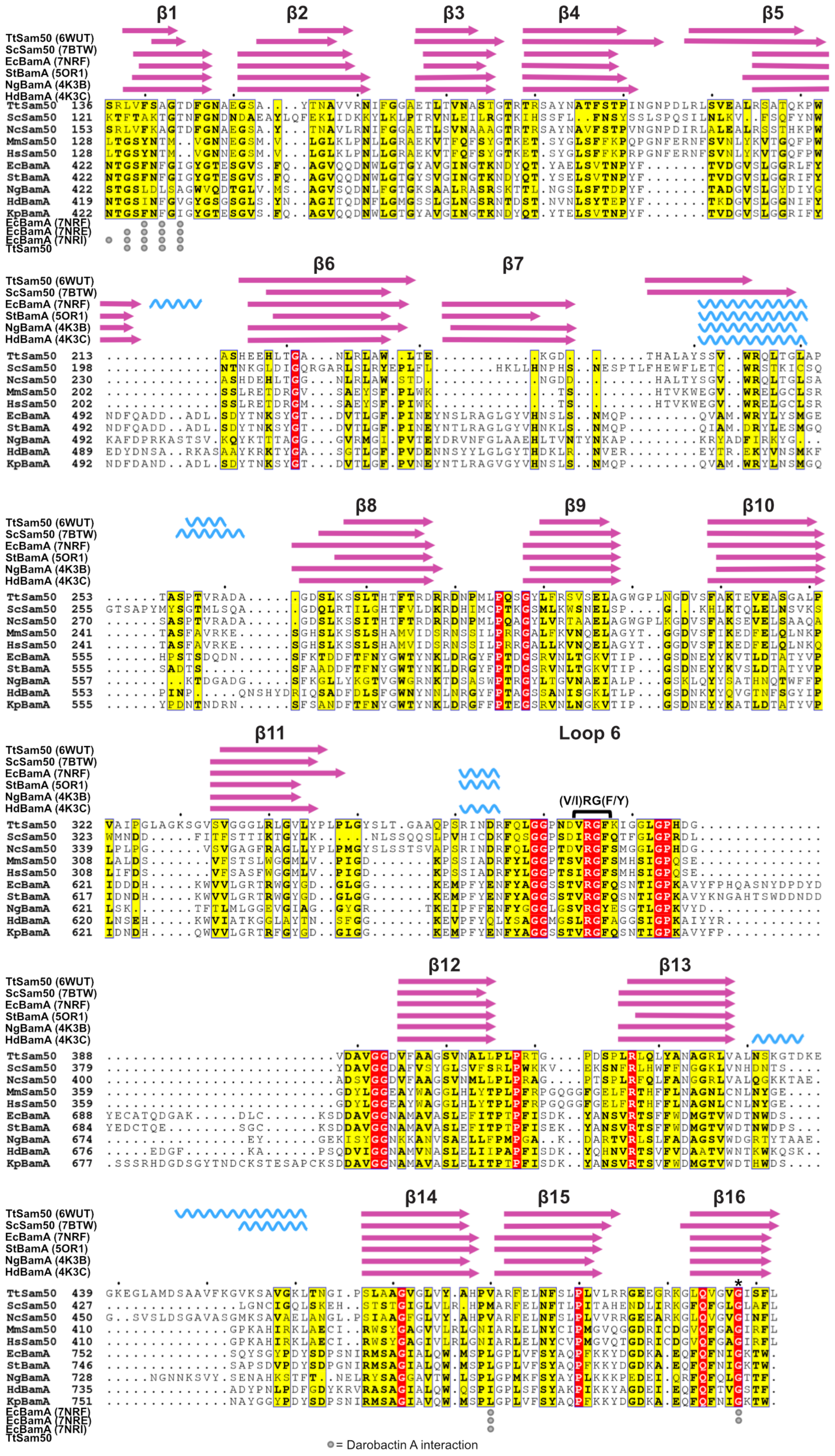

Supplementary Figure 2. Structure-coded sequence alignment of Sam50 and BamA β-barrels.

Magenta arrows represent β-strands, blue waves represent α-helices. Kinking glycine in β16 annotated with asterisk. Darobactin A binding interactions identified by PyMOL (Version 2.4 Schrödinger, LLC) analysis annotated as grey circles when applicable. Alignment was generated by T-Coffee Expresso [1–5], adjusted with JalView 2.11 [6] and colored with ESPript 3.0 [7] based on percent equivalent and 0.6 global score. Tt: *Thermothelomyces thermophilus* (UniProt G2QFF9; PDB 6WUT), Sc: *Saccharomyces cerevisiae* (UniProt P53969; PDB 7BTX), Nc: *Neurospora crassa* (UniProt V5IKW7), Mm: *Mus musculus* (UniProt Q8BGH2), Hs: *Homo sapiens* (UniProt Q9Y512), Ec: *Escherichia coli* (UniProt P0A940; PDB 7NRF, 7NRE, 7NRI), St: *Salmonella typhimurium* (UniProt Q8ZRP0; PDB 5OR1), Ng: *Neisseria gonorrhoeae* (UniProt Q5F5W8; PDB 4K3B), Hd: *Haemophilus ducreyi* (UniProt Q93PM2; PDB 4K3C), Kp: *Klebsiella pneumoniae* (UniProt B5Y1J4).

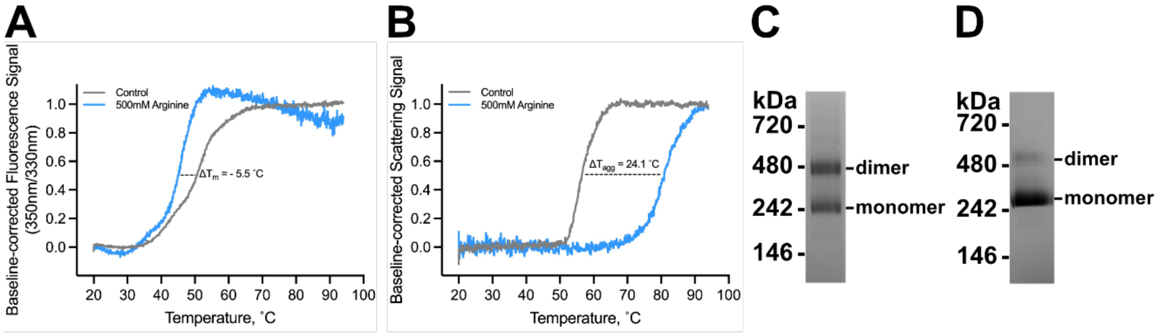

Supplementary Figure 3. Presence of arginine stabilizes monomeric SAM complex.

Ternary SAM complex (Sam50 (no tag), Sam35 (no tag), and TwinStrep-GG-Sam37) purified in LMNG was mixed with buffer additive screens, and sample thermostability measured and analyzed. (A-B) Representative baseline corrected thermal denaturation curves (A) and thermal aggregation curves (B) of the *Tt*SAM complex in the original purification buffer (grey) or with 500mM L-arginine added to purification buffer (blue). SAM complex in the presence of 500mM L-arginine has a reduced T_m, though steeper unfolding transition compared to control. Taken together with the large increase in T_{agg} observed in thermal aggregation plot, this data suggests that the presence of arginine is stabilizing for the SAM complex. (C) BN-PAGE of *Tt*SAM complex sample purified in LMNG (solubilization and strep affinity) and GDN (size exclusion chromatography) with no added arginine result in dimeric and monomeric populations. (D) BN-PAGE of *Tt*SAM complex purified with 200mM L-arginine added during LMNG solubilization and GDN affinity chromatography steps. Increase in monomer band intensity supports the thermal unfolding findings that addition of arginine stabilizes monomeric SAM complex. Source data are provided as a Source Data file.

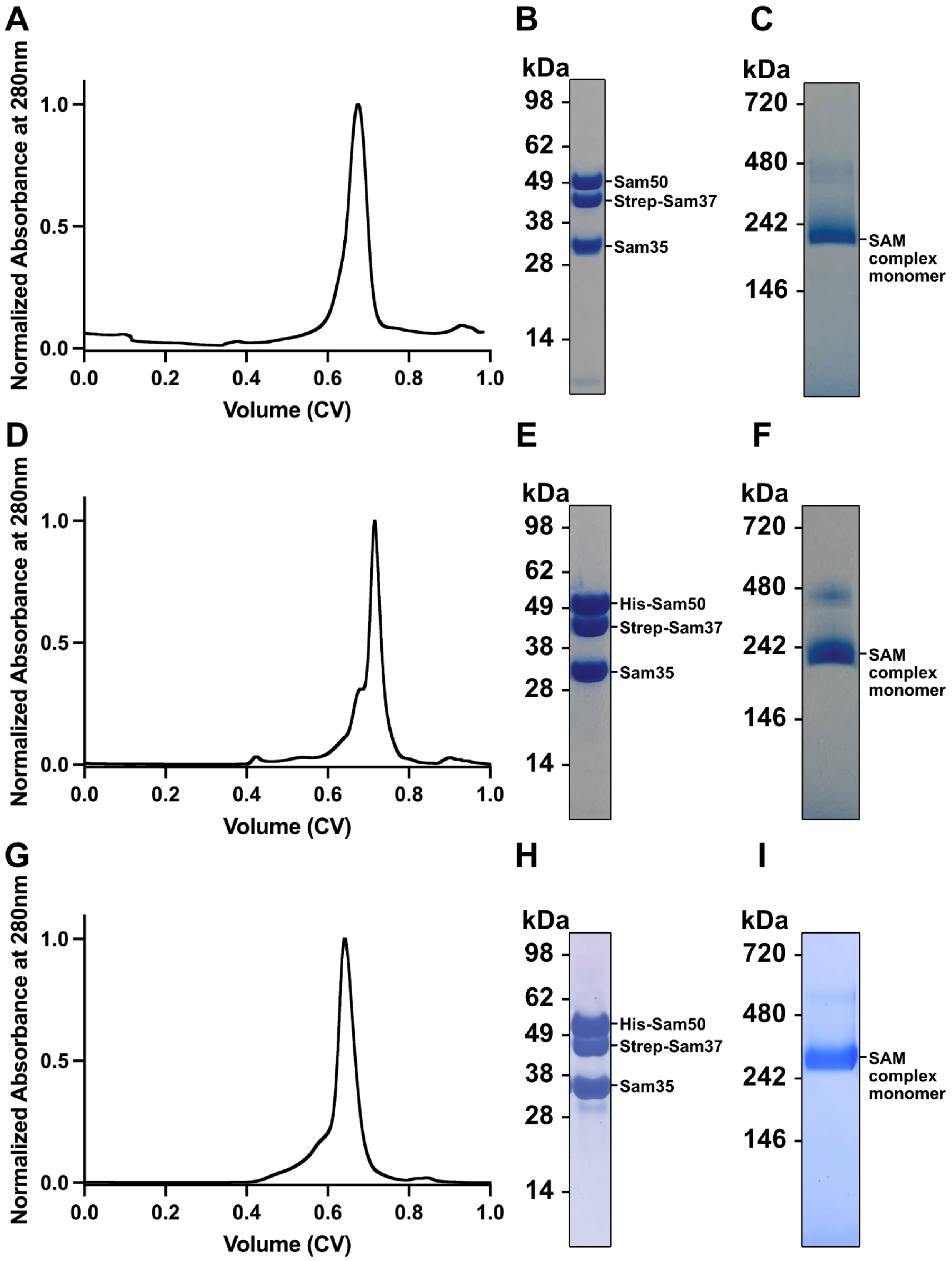

Supplementary Figure 4. Purification of *T. thermophilus* SAM complex in detergent.

(A) Representative size exclusion chromatogram of TwinStrep-tagged SAM complex purified in LMNG/GDN and used for structural studies with darobactin A. Peak fraction was subjected to (B) SDS-PAGE demonstrating all three subunits present and (C) BN-PAGE demonstrating primarily monomeric population. (D) Representative size exclusion chromatogram of His-tagged and strep-tagged SAM complex purified in LMNG/GDN for MST studies. Peak fraction was subjected to (E) SDS-PAGE demonstrating all three subunits present and (F) BN-PAGE demonstrating primarily monomeric population. (G) Representative size exclusion chromatogram of His-tagged and TwinStrep-tagged SAM complex purified in GDN and used for SAM structural studies. Peak fraction was subjected to (H) SDS-PAGE demonstrating all three subunits present and (I) BN-PAGE demonstrating primarily monomeric population. Data are representative of three independent experiments. Source data are provided as a Source Data file.

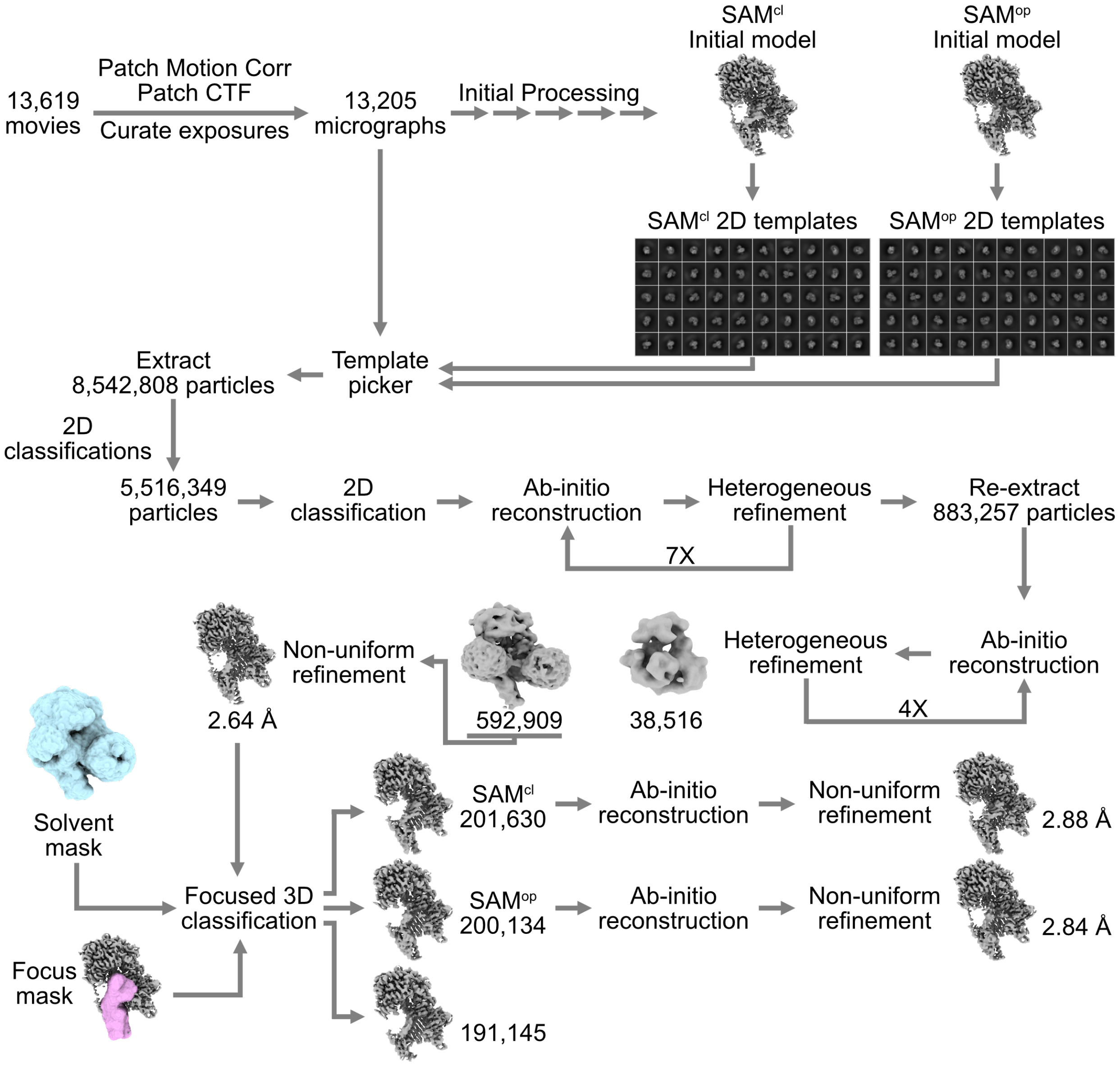

Supplementary Figure 5. Cryo-EM data processing workflow of the SAM complex in GDN.

Processing completed using cryoSPARC v4.4.0.

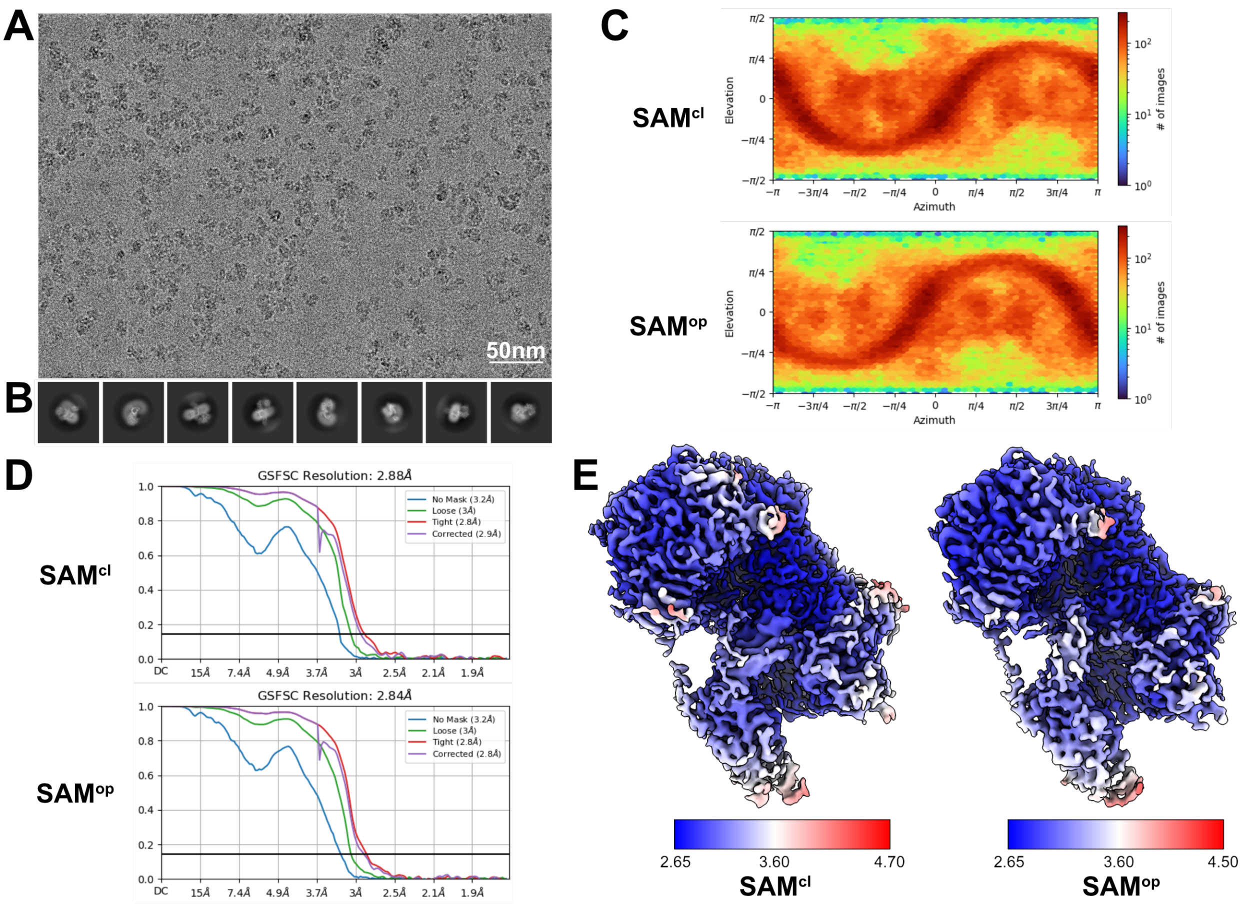

Supplementary Figure 6. Cryo-EM data of the SAM complex in GDN.

(A) Representative cryo-EM micrograph (top) and 2D classes (bottom). (B) Selected representative 2D classes from cryoSPARC processing. 360pix box size. (C) Orientation distribution plots for refined maps. (D) Fourier Shell Coefficient (FSC) curves for refined maps. (E) Sharpened maps colored by local resolution calculated in Phenix v1.19 [8].

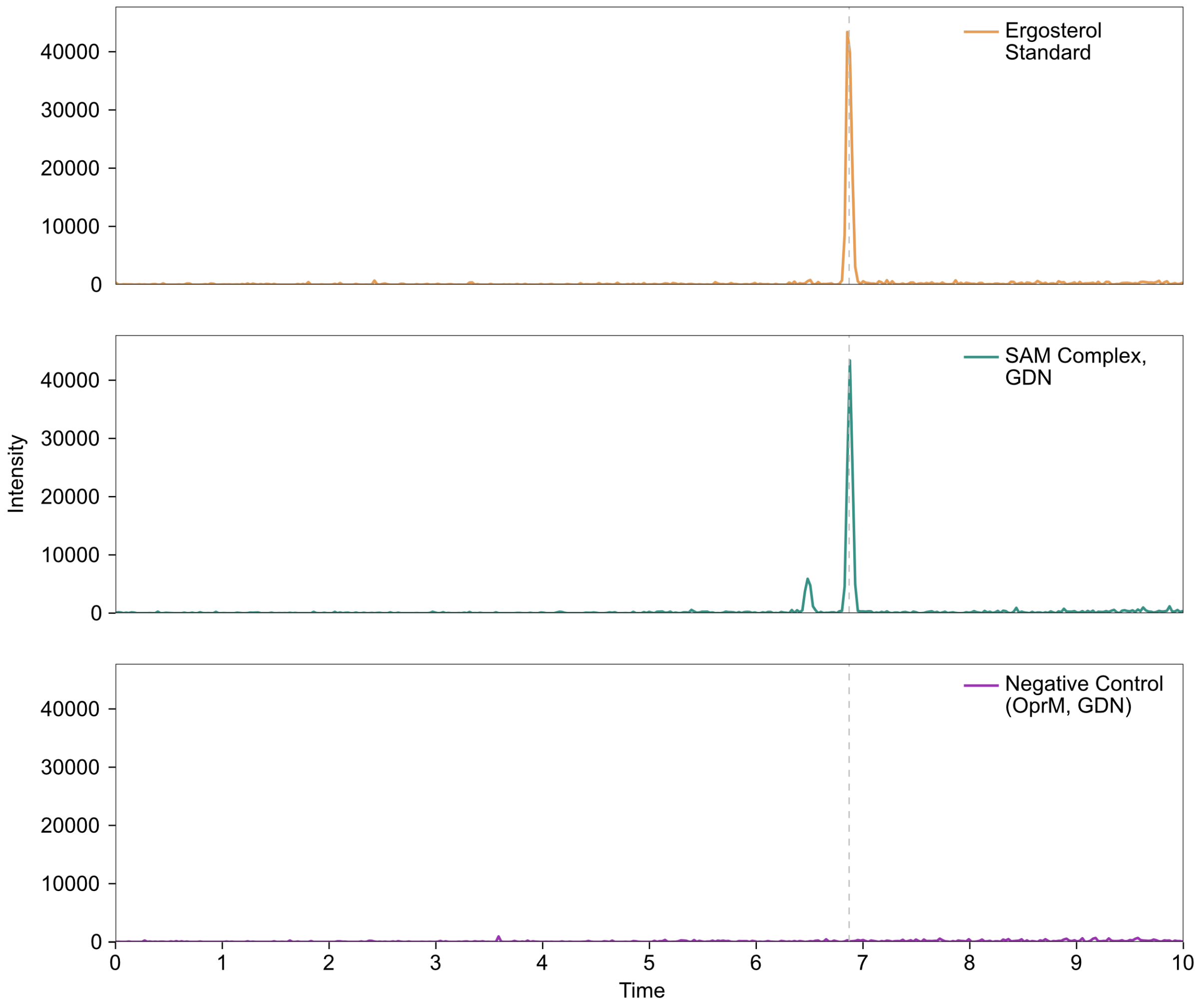

Supplementary Figure 7. LC/MS analysis of the SAM complex purified in GDN confirms presence of ergosterol. Representative chromatograms for ergosterol as measured by the parent daughter ion pair 379.6->171.4 using positive polarity. The representative standard injection contained 20 µM ergosterol. The grey dashed line in each chromatogram indicated the expected retention time based on the standard. The negative control sample, a bacterial OM β-barrel protein purified in GDN, does not contain ergosterol. See Source Data file for raw integrations.

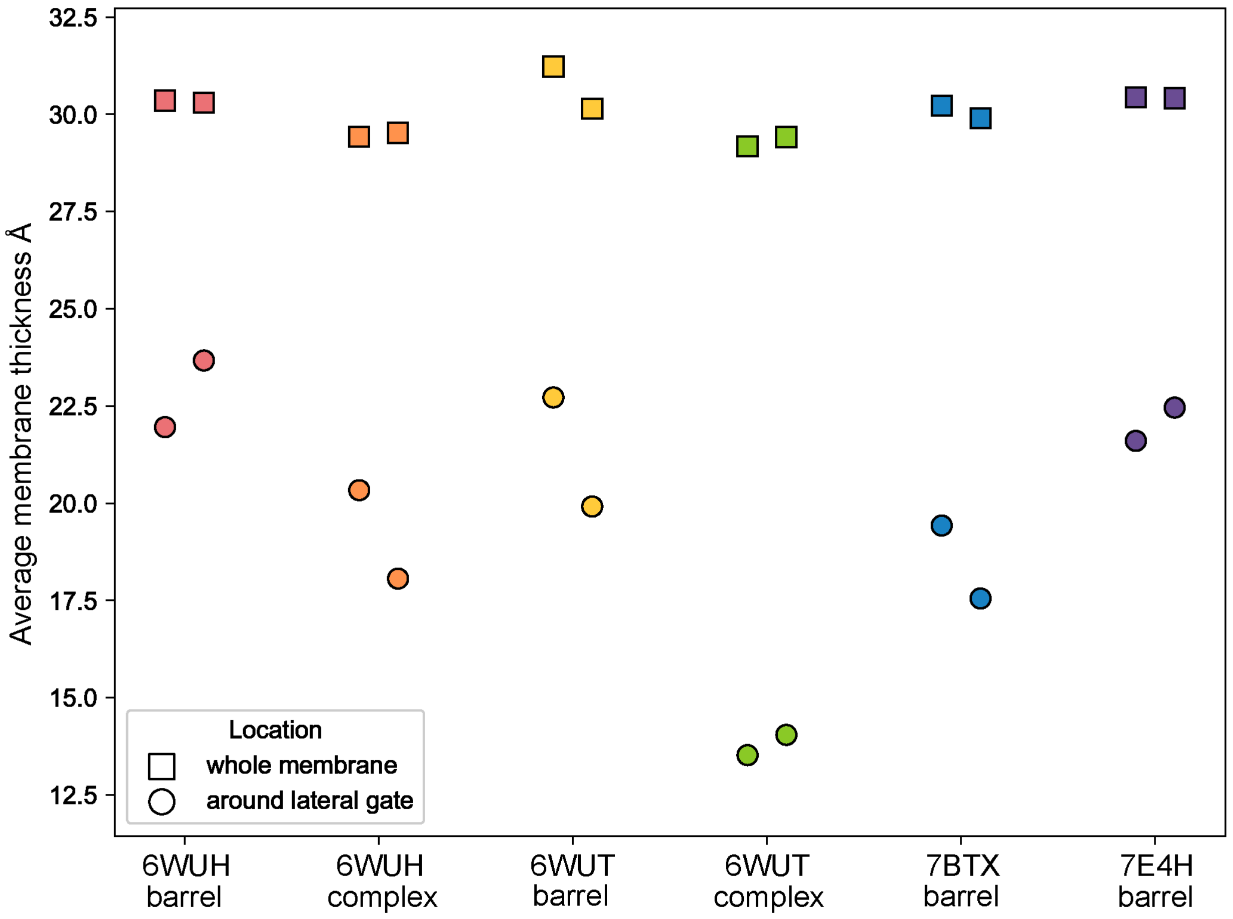

Supplementary Figure 8. Average membrane thickness measurements for different membrane embedded complexes, highlighting the distinct thinning near the lateral gate. Average membrane thickness, measured as the z-axis separation of lipid headgroups from the upper and lower leaflets, averaged for full membrane or specifically around the lateral gate, for the last 2 µs of each membrane embedded complex system. Dots represent the average thickness around the lateral gate, while squares indicate the average thickness across the entire membrane. Each dot or square corresponds to a single replica. Replicate data are in provided Source Data file.

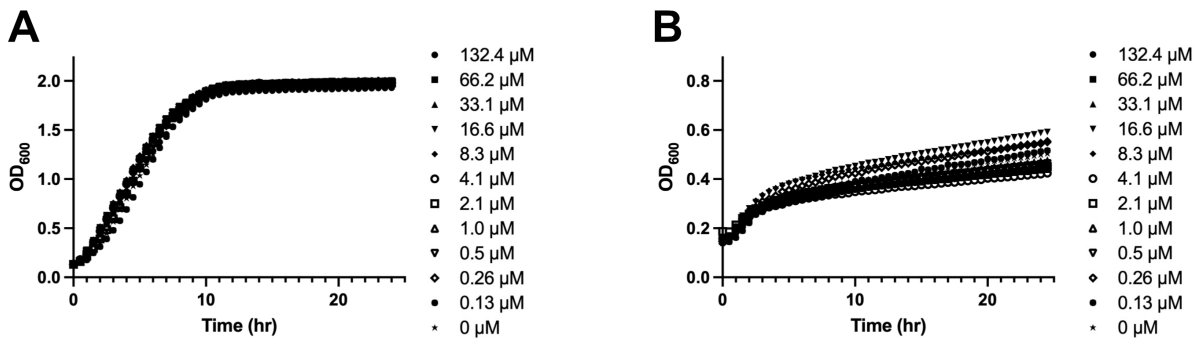

Supplementary Figure 9. Darobactin A does not inhibit *Saccharomyces cerevisiae* cell growth. Growth curves of *Saccharomyces cerevisiae* in the presence of 0-132.4µM darobactin A in (A) YPD or (B) YPG media. Plotted points are average of two technical replicates. Data are representative of two independent experiments. Replicate data are in provided Source Data file.

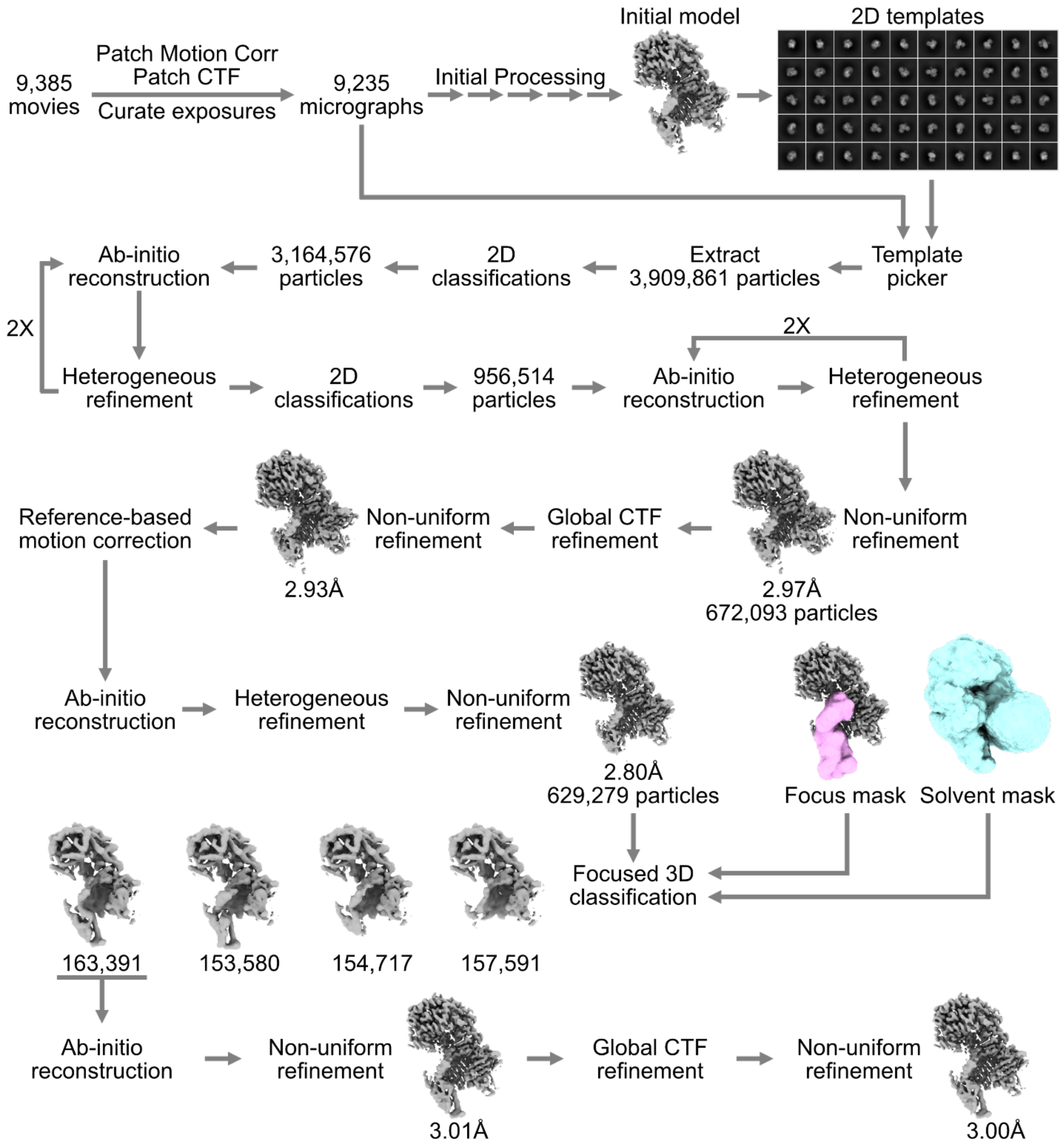

Supplementary Figure 10. Cryo-EM data processing workflow of the darobactin A bound SAM complex in detergent. Processing completed using cryoSPARC v4.4.1.

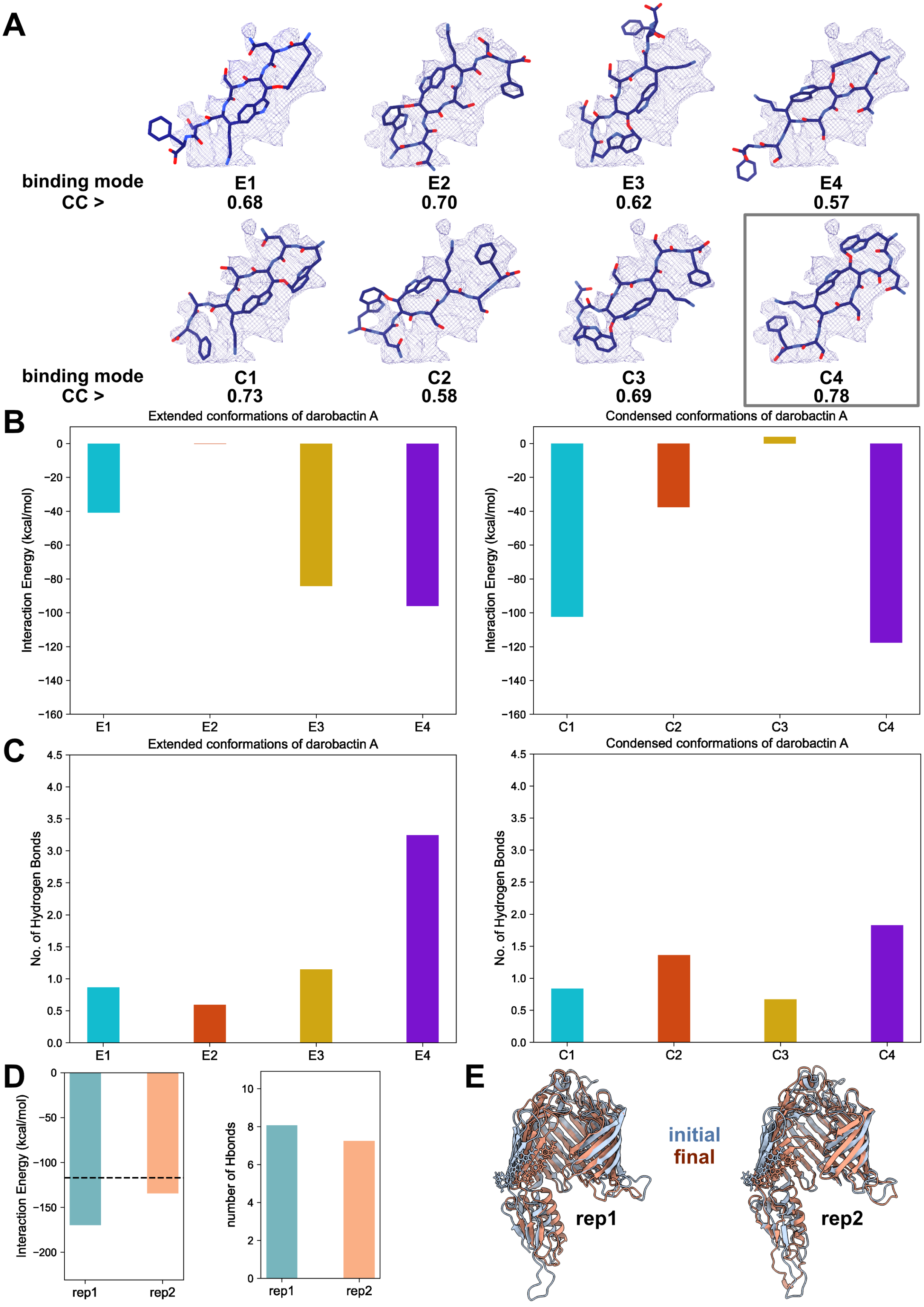
A
binding mode
CC >
E1
0.68
E2
0.70
E3
0.62
E4
0.57
binding mode
CC >
C1
0.73
C2
0.58
C3
0.69
C4
0.78
B
Extended conformations of darobactin A
Condensed conformations of darobactin A
Interaction Energy (kcal/mol)
C
Extended conformations of darobactin A
Condensed conformations of darobactin A
No. of Hydrogen Bonds
D
Interaction Energy (kcal/mol)
number of Hbonds
rep1
rep2
E
initial
final
rep1
rep2

Supplementary Figure 11. Determination of darobactin A orientation within the initial cryo-EM density. (A) Fit of Phenix refined darobactin A models (midnight blue) within density from initial cryo-EM map. Respective Phenix refinement CC score for darobactin A shown below each superposition of sharpened map and refined model. Top row contains the terminal phenyl group in an extended conformation (“E”) while bottom row contains a compact terminal phenyl group conformation (“C”). Grey box identifies the darobactin A orientation in the final structure, chosen based on refinement statistics, fit within density, and MDFF results. (B-C) Comparison of (B) interaction energy and (C) hydrogen bonds between darobactin A and Sam50 for different conformations of darobactin A. (D) Comparison of interaction energy (left) and number of hydrogen bonds formed (right) between darobactin A and Sam50 during the equilibration simulation of the darobactin-bound SAM complex for two independent replicas. The dotted line indicates the interaction energy calculated from the MDFF simulations based on the “C4” conformation. Energy and hydrogen bond counts are averaged over last 0.5 µs of the simulation. (E) The initial (blue) and final (salmon) conformations of the two independent replicas from the equilibration simulation of the darobactin-bound SAM complex. Sam50 depicted in cartoon representation, darobactin A in stick representation.

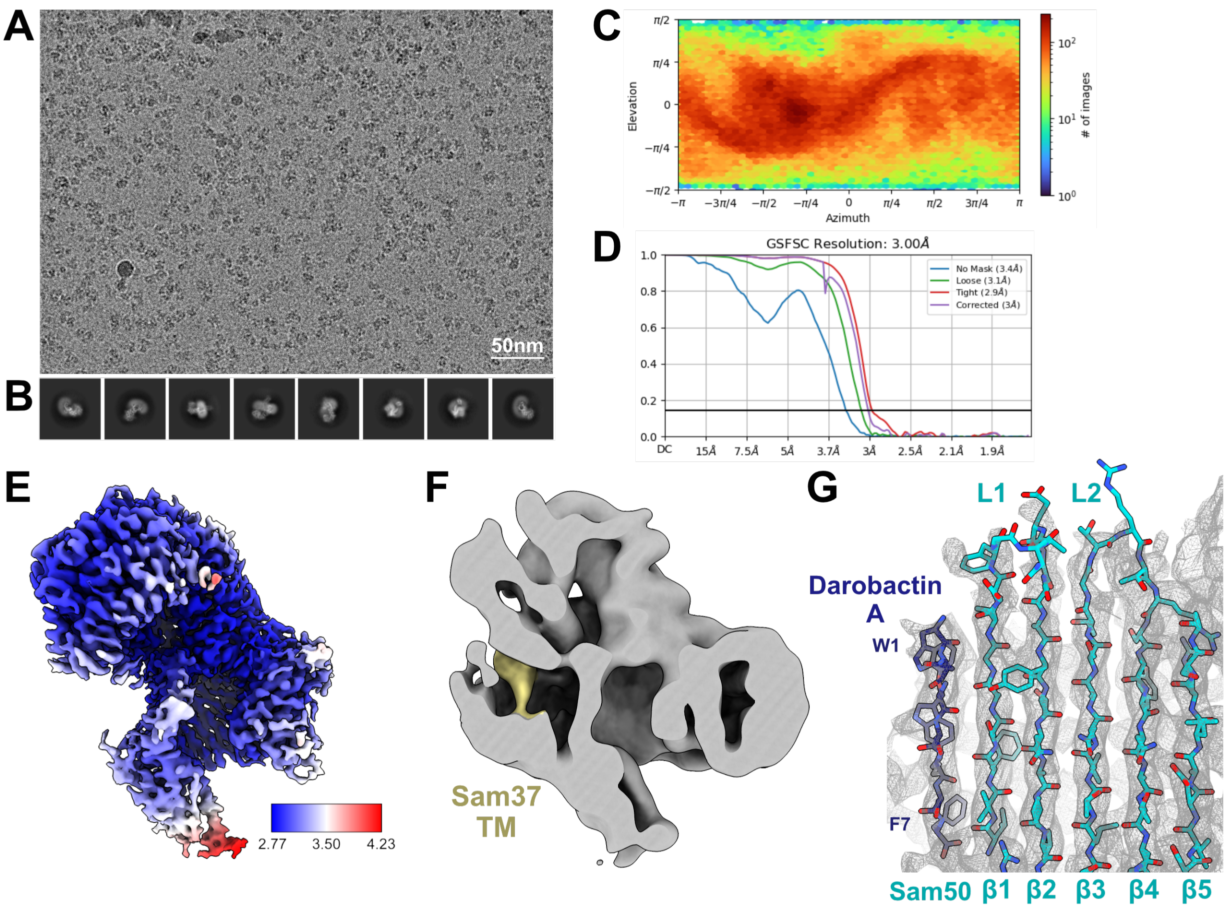

Supplementary Figure 12. Cryo-EM data of the darobactin A bound SAM complex in detergent. (A) Representative cryo-EM micrograph (B) Selected representative 2D classes from cryoSPARC processing. 360pix box size. (C) Orientation distribution plot for refined map. (D) Fourier Shell Coefficient (FSC) curves for refined map. (E) Sharpened map colored by local resolution calculated in Phenix v1.19 [8]. (F) Density of the Sam37 transmembrane helix is visible (yellow) when the cryo-EM density map is lowpass filtered to 10 Å. (G) Superposition of sharpened cryo-EM map (grey) and darobactin A (dark blue) and Sam50 β1-3 (dark turquoise) model in stick representation.

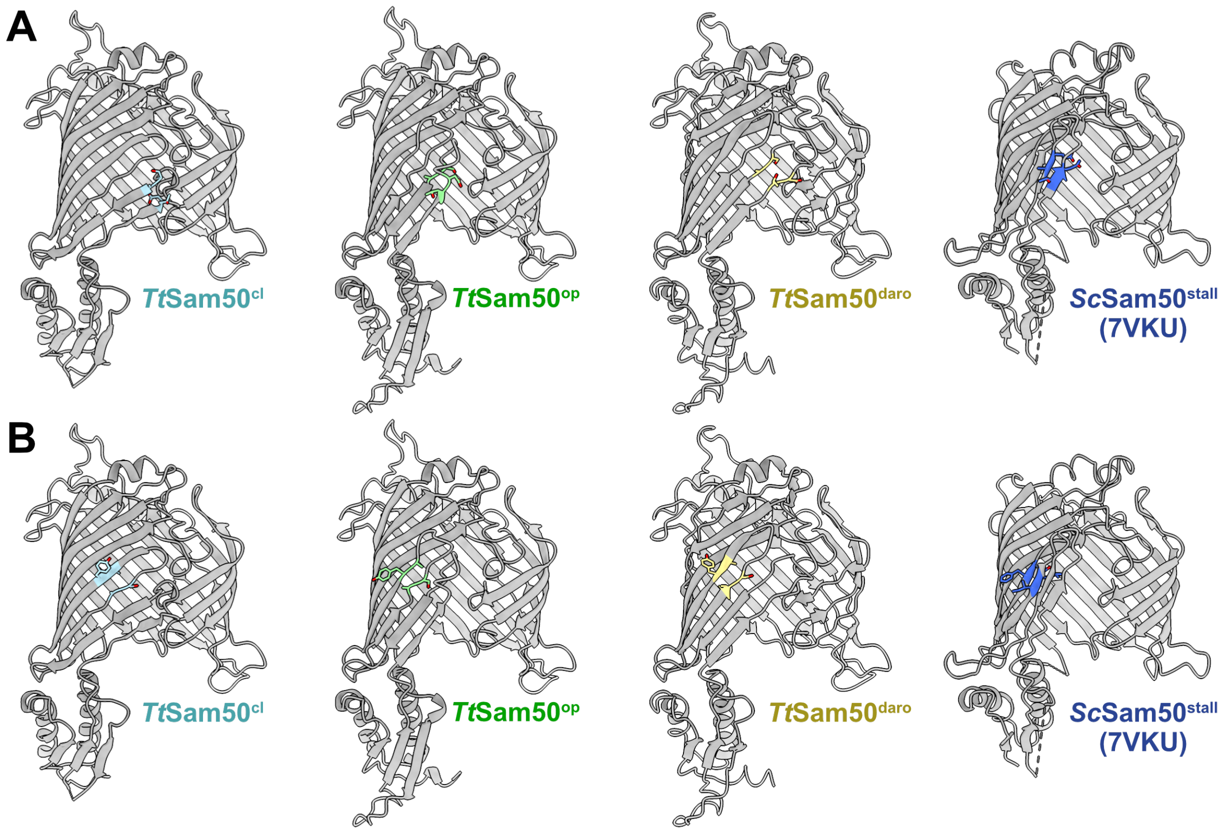

Supplementary Figure 13. Sam50 residues used in lateral gate opening measurements.

Cartoon representation of Sam50 structures are shown in gray. Residues used to determine extent of lateral gate opening in Table 2 are colored and shown in stick representation for (A) loop 1 and (B) loop 2. See Table 2 for distances of Sam50 lateral gate opening.

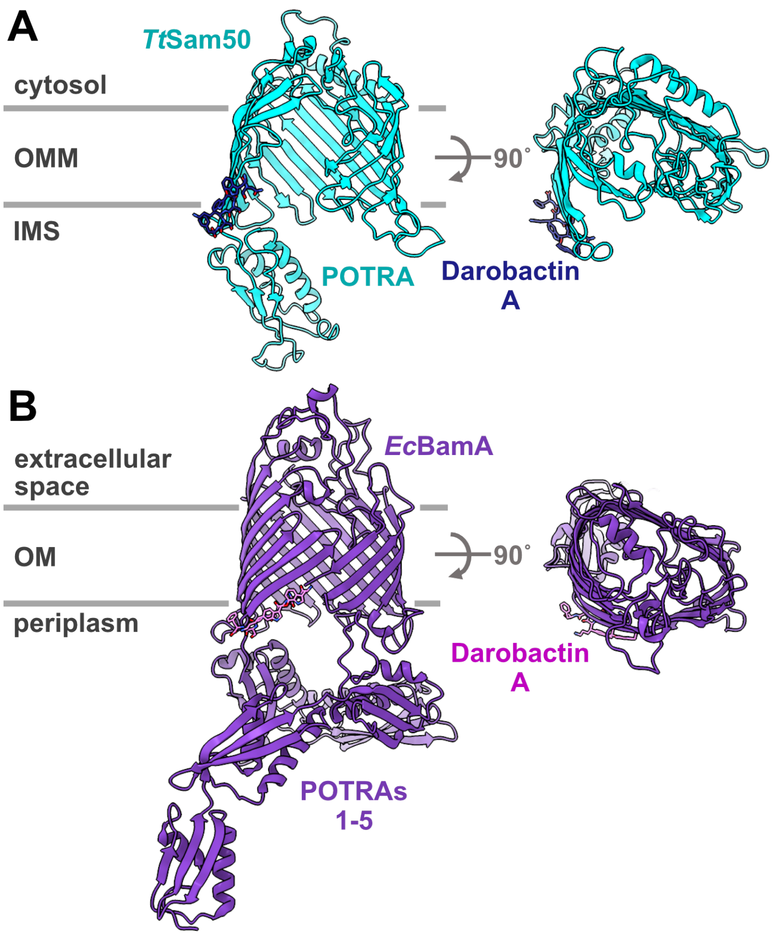

Supplementary Figure 14. Structural comparison of Sam50 and BamA bound to darobactin A.

(A) Side and top views of Sam50 (turquoise) bound to darobactin A (dark blue). (B) Side and top views of *E. coli* BamA (purple) bound to darobactin A (dark blue) (PDB: 7NRI). See also Figure 4F for superposition of these structures.

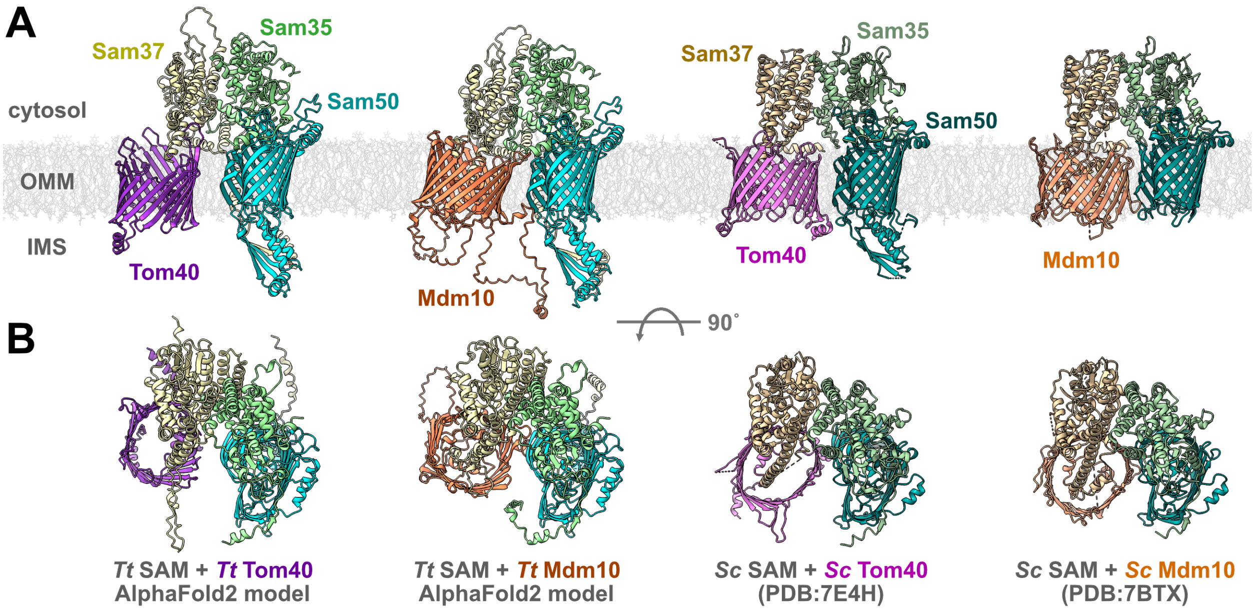

Supplementary Figure 15. Comparison of SAM complex AlphaFold models and experimental structures that contain a second β-barrel.

T.thermophilus SAM complex AlphaFold models and *S. cerevisiae* SAM complex structures (PDB ID: 7E4H, 7BTX) viewed from (A) the membrane plane and (B) from the top. AlphaFold v2.3.2 multimer was used for modeling the complexes [9,10]. Unstructured terminal regions were removed for clarity.

Peptide	Biological Replicate #	K_d (M)	± confidence (M)	Standard Error of Regression
Darobactin A	1	1.49E-07	5.67E-08	0.8323
	2	2.96E-07	3.13E-08	0.2935
	3	3.77E-07	6.38E-08	0.4485
	4	2.86E-07	7.66E-08	0.8661
	5	9.56E-08	4.38E-08	0.8250
	6	1.66E-07	7.47E-08	0.8690
Linear Darobactin	1	1.20E-03	2.38E-04	0.6623
	2	1.17E-03	4.56E-04	0.7701
	3	4.91E-04	2.56E-04	0.8838
*Tt*Tom40 β-signal peptide	1	2.44E-06	1.29E-06	2.4606
	2	4.85E-06	1.99E-06	1.3380
	3	1.97E-06	1.06E-06	0.6525
*Tt*Porin1 β-signal peptide	1	1.05E-05	3.07E-06	0.4325
	2	3.37E-06	7.84E-07	0.8100
	3	3.99E-06	1.60E-06	1.9277

Supplementary Table 1. Binding affinities of peptides to *Tt*SAM complex purified in detergent.

Source data are provided as a Source Data file.

**Name**	**Sequence**	**Molecular weight (Da)**	**Source**
Darobactin A		966.4	Lewis Lab
Linear Darobactin	WNWSKSF	954.1	GenScript
*Tt*Porin1 β-signal Peptide	DTQKLDQATHKVGTSFTFESC	2343.6	GenScript
*Tt*Tom40 β-signal Peptide	PPVMMTFAADVDHFTQQAKIGVGISIE	2903.4	GenScript

Supplementary Table 2. Peptides used in this study.

Porin1 and Tom40 β-signal peptides are designed based on *T. thermophilus* sequences. Linear darobactin contains the same amino acid sequence as darobactin A, but no backbone cyclizations.

	Sam35 (A16-A333)		Sam50 (B40-B512)		Sam37 (C1-C421)	
	Calpha	All	Calpha	All	Calpha	All
SAM^{daro} - SAM^{op}	0.72	1.09	1.54	1.85	0.86	1.23
SAM^{daro} - SAM^{cl}	0.73	1.10	2.79	2.95	0.87	1.29
SAM^{op} - SAM^{cl}	0.33	0.67	3.53	3.62	0.47	0.88

Supplementary Table 3. RMSD calculations

RMSD in Angstroms between overlapping chains, calculated in ccp4i.

Name	Species	UniProt ID
Sam50	*Thermothelomyces thermophilus*	G2QFF9
Sam35	*Thermothelomyces thermophilus*	G2QAT9
Sam37	*Thermothelomyces thermophilus*	G2Q6R7
Tom40	*Thermothelomyces thermophilus*	G2Q9I9
Porin1	*Thermothelomyces thermophilus*	G2Q8B8
Sam50	*Saccharomyces cerevisiae*	P53969
Tom40	*Saccharomyces cerevisiae*	P23644
Porin1	*Saccharomyces cerevisiae*	P04840
Mdm10	*Saccharomyces cerevisiae*	P18409

Supplementary Table 4. UniProt IDs of proteins used in this study.

Name	Promoter	Backbone	Expressed Protein	Source
*Tt*Sam50 pBEVY-GT	GAL1	pBEVY-GT	*Tt*Sam50	[11]
10xHis-GS-TEV-*Tt*Sam50 pBEVY-GT	GAL1	pBEVY-GT	10xHis-GS-TEV-*Tt*Sam50	[11]
*Tt*Sam35 pBEVY-GL	GAL1	pBEVY-GL	*Tt*Sam35	[11]
TwinStrep-GG-*Tt*Sam37 pBEVY-GU	GAL1	pBEVY-GU	TwinStrep-GG-*Tt*Sam37	[11]
TwinStrep-TEV-GG-*Tt*Sam37 pBEVY-GU	GAL1	pBEVY-GU	TwinStrep-TEV-GG-*Tt*Sam37	[11]
*Sc*Tom40 pGEM-4Z	SP6	pGEM-4Z	*Sc*Tom40	This study
*Sc*Sam50 pGEM-4Z	SP6	pGEM-4Z	*Sc*Sam50	This study
*Sc*Por1 pGEM-4Z	SP6	pGEM-4Z	*Sc*Porin1	This study
*Sc*Mdm10 pGEM-4Z	SP6	pGEM-4Z	*Sc*Mdm10	This study

Supplementary Table 5. List of plasmids used in this study.

Strain use	*Expression Plasmids*	*Genotype*
in vitro mitochondrial import assays, growth assays, transformed for SAM coexpressions	--	*MATα {leu2-3,112 trp1-1 can1-100 ura3-1 ade2-1 his3-11,15}*
Protein expression for buffer optimization experiments and SAM-darobactin A cryo-EM studies	*TtSam50 pBEVY-GT*	*MATα {leu2-3,112 trp1-1 can1-100 ura3-1 ade2-1 his3-11,15}*
	TtSam35 pBEVY-GL	
	TwinStrep-GG-TtSam37 pBEVY-GU	
Protein expression for MST studies, SAM monomer cryo-EM studies, and lipid LC/MS analyses	*10xHis-GS-TEV-TtSam50 pBEVY-GT*	*MATα {leu2-3,112 trp1-1 can1-100 ura3-1 ade2-1 his3-11,15}*
	TtSam35 pBEVY-GL	
	TwinStrep-TEV-G-TtSam37 pBEVY-GU	

Supplementary Table 6. *S. cerevisiae* strains used in this study.

Application	Product	Primer 1	Primer 2
Linearize DNA for *in vitro* transcription with SP6 promoter	SP6 promoter and Tom40, Sam50, Por1, or Mdm10	CCCAGTCACGACGTTGTAAAACG	GGAAACAGCTATGACCATG

Supplementary Table 7. List of primers used in this study.

Supplementary References

1. Armougom, F. *et al.* Expresso: Automatic incorporation of structural information in multiple sequence alignments using 3D-Coffee. *Nucleic Acids Res* 34, 604–608 (2006).
2. Di Tommaso, P. *et al.* T-Coffee: A web server for the multiple sequence alignment of protein and RNA sequences using structural information and homology extension. *Nucleic Acids Res* 39, 13–17 (2011).
3. Notredame, C., Higgins, D. G. & Heringa, J. T-coffee: A novel method for fast and accurate multiple sequence alignment. *J Mol Biol* 302, 205–217 (2000).
4. O'Sullivan, O., Suhre, K., Abergel, C., Higgins, D. G. & Notredame, C. 3DCoffee: Combining Protein Sequences and Structures within Multiple Sequence Alignments. *J Mol Biol* 340, 385–395 (2004).
5. Poirot, O., Suhre, K., Abergel, C., O'Toole, E. & Notredame, C. 3DCoffee@igs: a web server for combining sequences and structures into a multiple sequence alignment. *Nucleic Acids Res* 32, W37–W40 (2004).
6. Waterhouse, A. M., Procter, J. B., Martin, D. M. A., Clamp, M. & Barton, G. J. Jalview Version 2-A multiple sequence alignment editor and analysis workbench. *Bioinformatics* 25, 1189–1191 (2009).
7. Robert, X. & Gouet, P. Deciphering key features in protein structures with the new ENDscript server. *Nucleic Acids Res* 42, 320–324 (2014).
8. Liebschner, D. *et al.* Macromolecular structure determination using X-rays, neutrons and electrons: recent developments in Phenix. *Acta Crystallogr D Struct Biol* 75, 861–877 (2019).
9. Jumper, J. *et al.* Highly accurate protein structure prediction with AlphaFold. *Nature* 596, 583–589 (2021).
10. Evans, R. *et al.* Protein complex prediction with AlphaFold-Multimer. *bioRxiv* (2022) doi:10.1101/2021.10.04.463034.
11. Diederichs, K. A. *et al.* Structural insight into mitochondrial β-barrel outer membrane protein biogenesis. *Nat Commun* 11, 3290 (2020).
