# Supplementary material for: The dynamic lateral gate of the mitochondrial β-barrel biogenesis machinery is blocked by darobactin A
Source: Nat Commun. 2025 Nov 20;16:11349. doi: 10.1038/s41467-025-66417-0 (PMC12728192; doi:10.1038/s41467-025-66417-0)
Supplement: Supplementary file 2 — Description of Additional Supplementary Files [file 41467_2025_66417_MOESM2_ESM.pdf]

Description of Additional Supplementary Files

Supplementary Movie 1. Sam50 lateral gate dynamics observed in morph between SAM^{cl} and SAM^{op} cryo-EM structures.

Supplementary Movie 2. This movie illustrates the dynamic behavior of the Sam50 protein simulated in isolation, without other components of the SAM complex. The visualization captures the transition of Sam50 from an open to a closed conformation.

Supplementary Movie 3. This movie illustrates the dynamics of the 6WUH-complex, highlighting the interaction of a DOPC molecule (depicted in red, Van der Waals representation) as it approaches the lateral gate of the complex. The visualization demonstrates the molecule's transient interaction with the gate, without entering the lumen.
