# Supplementary material for: The dynamic lateral gate of the mitochondrial β-barrel biogenesis machinery is blocked by darobactin A
Source: Nat Commun. 2025 Nov 20;16:11349. doi: 10.1038/s41467-025-66417-0 (PMC12728192; doi:10.1038/s41467-025-66417-0)
Supplement: Supplementary file 6 — Reporting Summary [file 41467_2025_66417_MOESM6_ESM.pdf]

nature portfolio

Corresponding author(s): Joseph A. Mindell
Susan K. Buchanan

Last updated by author(s): 10/29/2025

Reporting Summary

Nature Portfolio wishes to improve the reproducibility of the work that we publish. This form provides structure for consistency and transparency in reporting. For further information on Nature Portfolio policies, see our Editorial Policies and the Editorial Policy Checklist.

Statistics

For all statistical analyses, confirm that the following items are present in the figure legend, table legend, main text, or Methods section.

n/a	Confirmed	
x		The exact sample size (*n*) for each experimental group/condition, given as a discrete number and unit of measurement
	x	A statement on whether measurements were taken from distinct samples or whether the same sample was measured repeatedly
x		The statistical test(s) used AND whether they are one- or two-sided<br>*Only common tests should be described solely by name; describe more complex techniques in the Methods section.*
x		A description of all covariates tested
x		A description of any assumptions or corrections, such as tests of normality and adjustment for multiple comparisons
	x	A full description of the statistical parameters including central tendency (e.g. means) or other basic estimates (e.g. regression coefficient) AND variation (e.g. standard deviation) or associated estimates of uncertainty (e.g. confidence intervals)
x		For null hypothesis testing, the test statistic (e.g. *F*, *t*, *r*) with confidence intervals, effect sizes, degrees of freedom and *P* value noted<br>*Give P values as exact values whenever suitable.*
x		For Bayesian analysis, information on the choice of priors and Markov chain Monte Carlo settings
x		For hierarchical and complex designs, identification of the appropriate level for tests and full reporting of outcomes
x		Estimates of effect sizes (e.g. Cohen's *d*, Pearson's *r*), indicating how they were calculated

Our web collection on statistics for biologists contains articles on many of the points above.

Software and code

Policy information about availability of computer code

Data collection	Prometheus NT.48 (NanoTemper Technologies), Monolith NT.115 (NanoTemper Technologies), Fujifilm FLA-9000 Phosphorimager, ClarioStar Plus plate reader, SerialEM
Data analysis	MoltenProt, cryoSPARC (versions 4.4.0 and 4.4.1), Chimera (version 1.15), Coot, Phenix (versions 1.20 and 1.21), Isolde (version 1.8), QT PISA, PyMOL (version 2.4.1), ChimeraX (versions 1.4 and 1.7), CCP4i (version 8), MO.Affinity Analysis (NanoTemper Technologies), GraphPad Prism 9, AlphaFold2, SWISS-MODEL, LOOS package, Visual Molecular Dynamics (VMD) 1.9.4a57, NAMD 3.0, T-Coffee Expresso, JalView (version 2.11), ESPript 3.0, SciexOS 3.1 (AB Sciex Pte. Ltd.)

For manuscripts utilizing custom algorithms or software that are central to the research but not yet described in published literature, software must be made available to editors and reviewers. We strongly encourage code deposition in a community repository (e.g. GitHub). See the Nature Portfolio guidelines for submitting code & software for further information.

Data

Policy information about availability of data

All manuscripts must include a data availability statement. This statement should provide the following information, where applicable:

- Accession codes, unique identifiers, or web links for publicly available datasets
- A description of any restrictions on data availability
- For clinical datasets or third party data, please ensure that the statement adheres to our policy

Atomic coordinates and associated cryoEM 3D maps for the structures described here have been deposited in the wwPDB and EMDB under accession codes 9NK6/

EMD-49494 (SAMcl), 9NK7/EMD-49495 (SAMop), and 9NK8/EMD-49496 (SAMdaro). The full gel images, raw intensities for ergosterol MS measurements, and replicate data are provided in the Source Data file. Additional source data for all figures and files is available from the authors upon request.

Research involving human participants, their data, or biological material

Policy information about studies with human participants or human data. See also policy information about sex, gender (identity/presentation), and sexual orientation and race, ethnicity and racism.

Reporting on sex and gender	N/A
Reporting on race, ethnicity, or other socially relevant groupings	N/A
Population characteristics	N/A
Recruitment	N/A
Ethics oversight	N/A

Note that full information on the approval of the study protocol must also be provided in the manuscript.

Field-specific reporting

Please select the one below that is the best fit for your research. If you are not sure, read the appropriate sections before making your selection.

[x] Life sciences [ ] Behavioural & social sciences [ ] Ecological, evolutionary & environmental sciences

For a reference copy of the document with all sections, see nature.com/documents/nr-reporting-summary-flat.pdf

Life sciences study design

All studies must disclose on these points even when the disclosure is negative.

Sample size	Sample size was not predetermined. Microscope time was the limiting factor for cryoEM data collection, and therefore data was collected until the density resolution was sufficient for accurate model building. For biochemical and biophysical experiments, data was collected on 2-3 biological replicates were used, which is standard in the field. For mass spec experiments of a purified protein against a standard curve for quantification of a single molecule, a single injection was deemed appropriate to determine presence and suggest stoichiometry.
Data exclusions	MST experiments: Fluorescence and aggregation outliers were identified by MO.Control software and were removed prior to analysis. Faction bound datap oints with decreased signal at high peptide concentrations were excluded during data analysis for Kd determination, as the decreased signal at high peptide concentrations is inconsistent with a single binding curve and may reflect another process happening at these elevated concentrations. CryoEM data processing results in exclusion of poor quality particles. Standard protocols were followed for cryoEM data processing in CryoSPARC.
Replication	Two to three independent biological replicates were conducted for all biochemical and biophysical experiments.
Randomization	Randomization was not used, and is not applicable to this study.
Blinding	Blinding was not used, and is not applicable to this study.

Reporting for specific materials, systems and methods

We require information from authors about some types of materials, experimental systems and methods used in many studies. Here, indicate whether each material, system or method listed is relevant to your study. If you are not sure if a list item applies to your research, read the appropriate section before selecting a response.

Materials & experimental systems

n/a	Involved in the study	
x		Antibodies
	x	Eukaryotic cell lines
x		Palaeontology and archaeology
x		Animals and other organisms
x		Clinical data
x		Dual use research of concern
x		Plants

Methods

n/a	Involved in the study	
x		ChIP-seq
x		Flow cytometry
x		MRI-based neuroimaging

Eukaryotic cell lines

Policy information about cell lines and Sex and Gender in Research

Cell line source(s)	Saccharomyces cerevisiae strain W303.1B (MATα {leu2-3,112 trp1-1 can1-100 ura3-1 ade2-1 his3-11,15})
Authentication	Cell line was used for heterologous protein expression. No authentication was preformed, except for the use of selection markers.
Mycoplasma contamination	N/A
Commonly misidentified lines (See ICLAC register)	N/A

Plants

Seed stocks	N/A
Novel plant genotypes	N/A
Authentication	N/A
